# Analysis of Task Demand Effects on Visual and Auditory Mismatch Negativity (MMN) Across Autistic and Schizotypal Traits

**DOI:** 10.1111/ejn.70490

**Published:** 2026-04-10

**Authors:** Prune Mazer, Rita Pasion, Zohra Kamran Rao, Celeste Silveira, Fernando Ferreira‐Santos

**Affiliations:** ^1^ E2S Polytechnic Institute of Porto Porto Portugal; ^2^ Laboratory of Neuropsychophysiology, Faculty of Psychology and Education Sciences University of Porto Porto Portugal; ^3^ Faculty of Medicine University of Porto Porto Portugal; ^4^ HEI‐Lab—Digital Human‐Environment Interaction Labs Lusófona University Porto Portugal; ^5^ Faculty of Medicine Ivane Javakhishvili Tbilisi State University Tbilisi Georgia; ^6^ Psychiatry Department Centro Hospitalar Universitário São João Porto Portugal

**Keywords:** autistic traits, EEG/ERP, mismatch negativity (MMN), predictive processing, schizotypy

## Abstract

Mismatch negativity (MMN) is an event‐related potential component elicited by violations of sensory predictions and is widely interpreted, within the predictive processing framework, as a neural correlate of prediction error. Disruptions in prediction error signalling have been proposed as a potential mechanism underlying the diverse cognitive and perceptual profiles observed in autism and schizophrenia spectrum conditions. In this study, 122 community participants completed auditory and visual oddball tasks with two levels of target detection difficulty while undergoing EEG recording, alongside self‐report measures of autistic and schizotypal traits. We found that increased task difficulty significantly reduced MMN amplitude in both modalities, with large effect sizes for auditory (*d* = 1.826) and visual (*d* = 1.005) MMN, indicating a modulation by perceptual load. Although associations between MMN amplitude and trait dimensions were limited, emerging patterns suggest a potential dissociation between social and nonsocial autistic traits. These findings address key gaps in the literature, particularly the underrepresentation of visual MMN, and highlight the importance of multidimensional, cross‐modal approaches to investigate prediction error mechanisms in neurodiverse populations.

## Graphical abstract

Task difficulty reduced mismatch negativity (MMN) amplitude across auditory and visual modalities. Autistic traits showed modality and difficulty‐specific patterns: communication difficulties related to smaller auditory MMN in the easy task, while restricted interests/detail orientation related to larger visual MMN in the difficult task. We found no significant associations between MMN amplitude and schizotypal traits.

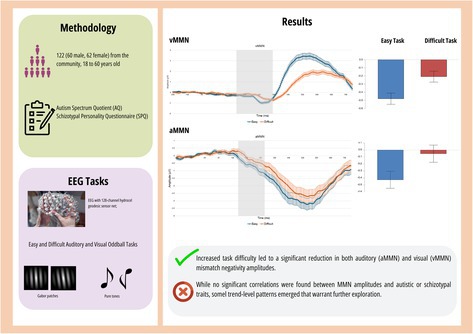

**Abbreviations**
ADHD: Attention‐deficit/hyperactivity disorder
aMMN: Auditory mismatch negativity
AQ: Autism‐spectrum quotient
ASD: Autism spectrum disorder
EEG: Electroencephalography
ERP/ERPs: Event‐related potential(s)
Fz: Frontal midline (EEG electrode site)
MMN: Mismatch negativity
PPF: Predictive processing framework
Pz: Parietal midline (EEG electrode site)
RIDO: Restricted interest and detail orientation
ROI: Region of interest
SPQ: Schizotypal personality questionnaire
SSD: Schizophrenia spectrum disorder
vMMN: Visual mismatch negativity

## 1 Introduction

The key component of event‐related potentials (ERPs) that measures brain activity when an unexpected stimulus disrupts a series of regular ones is the so‐called mismatch negativity (MMN). This neural indicator has been recognized for its relevance to the study of the brain's predictive processing mechanisms and is often used as a signal to index automatic, perceptual, and pre‐attentive processing (Näätänen et al. 2007). MMN is particularly compelling as it appears to occur independently of the person's level of alertness. This makes this component especially valuable not only for cognitive research but also for clinical applications (Garrido et al. 2009).

Within the predictive processing framework (PPF), MMN is considered a neural correlate of prediction error, since it is elicited by sensory signals that deviate from top–down expectations (Friston 2005; Garrido et al. 2009; Wacongne et al. 2011). In cognitive research, the oddball paradigm is one of the most established methods for eliciting MMN. In this task, frequent standard stimuli are presented repeatedly, engaging the brain's tendency to generate predictions. Less frequent deviant stimuli—that differ in one or more physical features from the standard stimuli—are then interspersed among the frequent stimuli. When a deviant stimulus occurs, it violates expectations, triggering a prediction error that prompts an update to the brain's predictive model (Garrido et al. 2009; Korka et al. 2022; Wacongne et al. 2011). In this sense, MMN offers a unique window of brain activity to explore predictive processing. For instance, task manipulations of the classic oddball paradigm, such as increasing uncertainty (Sapey‐Triomphe et al. 2025), can provide insights into how prediction error is modulated in the auditory and visual systems at early stages of perception. In such paradigms, the auditory MMN (aMMN) and visual MMN (vMMN) are elicited, and changes in their amplitude can serve as neural indicators of predictive model updates, with greater amplitude reflecting larger changes from expected patterns (see Garrido et al. 2009, for a detailed theoretical account).

An informative task manipulation that allows for exploring this possibility involves manipulating the task uncertainty by increasing or decreasing the perceptual difference between target and standard stimuli or manipulating the task difficulty. If the MMN amplitude reflects a prediction error, then larger deviations from the expected pattern might be associated with larger MMN amplitudes, as the size of the violation between the internal model and the input information increases (Lieder et al. 2013). Consistent with this view, studies have shown that larger perceptual differences between target and standard stimuli are often associated with increased aMMN amplitudes (see Näätänen et al. 2007 for a review). However, this relationship is not always straightforward. For instance, Horváth et al. (2008) demonstrated that increases in physical differences (20‐ to 320‐Hz frequency changes) led to shorter MMN latencies but not a monotonic increase in amplitude, suggesting that stimulus discriminability and overlap with the N1 response are critical parameters shaping this effect. Conversely, studies reviewed by Ghani et al. (2020) indicate that task difficulty manipulations, such as increasing cognitive workload with simultaneous visual tasks, do not consistently alter MMN amplitude, underscoring its relative independence from attentional demands. In addition, a meta‐analysis (Sapey‐Triomphe et al. 2025) showed that differences between individuals with ASD and neurotypical controls are more likely to emerge in task designs that increase uncertainty.

In the case of the vMMN, findings are more mixed. While some studies report a decrease in vMMN amplitude as the difficulty of detecting the deviant increases (Pazo‐Alvarez et al. 2004), results have been inconsistent (Kimura and Takeda 2013; Kremlacek et al. 2013). A recent meta‐analysis by Male (2025) found that effects of deviance magnitude on vMMN are more reliably detected for orientation deviants (e.g., Astikainen et al. 2008) but do not generalize across other types of visual deviation, such as spatial frequency (e.g., Maekawa et al. 2009). In addition, the studies included in this meta‐analysis often relied on small sample sizes, with an average of only 17 participants per study. As the author notes, such limited sample sizes can undermine the reliability of findings, leading to low replication rates, inflated effect sizes when results are significant, and limited predictive validity (Button et al. 2013). This is particularly important when exploring the possibility of using MMN in clinical practice.

In clinical research, MMN has become a valuable component for investigating predictive processing alterations across various neurodevelopmental and psychiatric conditions, including schizophrenia spectrum disorders (SSDs) and autism‐spectrum disorders (ASD) (Merchie and Gomot 2023). These conditions are frequently conceptualized as being at opposite ends of the PPF spectrum (Andersen 2022; Crespi and Badcock 2008; Tarasi et al. 2022). Individuals on the autism spectrum are theorized to rely less on prior expectations and to place greater weight on incoming sensory input, even when that input is noisy and should not be trusted (van de Cruys et al. 2014). This imbalance leads to poorly adjusted expectations and an increased registration of deviations as surprising, generating a heightened stream of prediction errors (Pellicano and Burr 2012; van de Cruys et al. 2014). Considering the MMN is thought to represent a prediction error (Wacongne et al. 2012), then it can be suggested that these individuals will show heightened MMN responses in tasks that violate expectation, such as oddball tasks. This process is thought to affect the overall information processing, rather than a single domain (e.g., social input), which could explain the heterogeneity of symptoms in ASD, including both social and nonsocial dimensions (Qela et al. 2025; van de Cruys et al. 2014). This framework suggests that this increased MMN pattern should not be exclusive to a single sensory modality but can also be found in other domains and with nonsocial stimuli. Supporting this, behavioral studies indicate that individuals with ASD or higher autistic traits often show superior performance in detail‐oriented tasks (Happé and Frith 2006; Cribb et al. 2016), enhanced abilities such as perfect pitch (Stanutz et al. 2014), and reduced susceptibility to visual illusions (Karvelis et al. 2018). At the same time, the same sensory‐driven processing style has been linked to hypersensitivity and a preference for routines (Lord et al. 2018).

In contrast, SSD may involve overly rigid or dominant prior beliefs that override incoming sensory evidence, resulting in reduced prediction error signals and diminished MMN amplitudes—patterns that may contribute to positive symptoms such as delusions and hallucinations (Sterzer et al. 2018). In this framework, internal expectations dominate perception, sometimes leading to false inferences, such as perceiving speech or faces in ambiguous stimuli (Blain et al. 2020; Corlett et al. 2019). Thus, while reduced reliance on priors in ASD may amplify prediction error responses, overreliance on priors in SSD may result in a reduction of these responses. This contrast substantiates the view that ASD and SSD represent opposite ends of a predictive processing spectrum, particularly along the autism–positive schizotypy axis, where nonsocial autistic traits such as detail‐focused reasoning stand in contrast to the heightened influence of prior beliefs seen in positive schizotypy (Andersen 2022; Zhang et al. 2025). As such, MMN provides a noninvasive, temporally accurate probe to compare different predictive processing profiles across these spectra. Meta‐analytical work has indeed uncovered intricate patterns of MMN modifications in clinical ASD and SSD.

In the case of ASD, although some individual studies using auditory and visual oddball paradigms reported differences between individuals with ASD and neurotypical controls, meta‐analytic findings did not support significant group differences in either sensory modality (Vassall et al. 2025). However, the majority of included studies in the meta‐analysis focused on pediatric samples and often lacked adequate age matching. This is a critical limitation, as even small age differences in childhood can introduce substantial variability in neurophysiological measures, potentially masking true group effects (Vassall et al. 2025). Among studies focusing on adult samples, findings remain mixed and mostly focus on auditory perception.

Two studies found overall reduced aMMN amplitudes in ASD in response to speech stimuli (Kujala et al. 2005) and also for emotional syllable and nonvocal sounds (Fan and Cheng 2014). On the other hand, three studies reported an opposite effect with overall increased aMMN between ASD and neurotypical groups, with two studies reporting this effect with pure tones (Haigh et al. 2022; Kujala et al. 2007) and one using speech stimuli (Matsuzaki et al. 2019). Some studies have also reported no overall group differences in aMMN amplitude between individuals with ASD and neurotypical controls but rather task‐related interaction effects. Goris et al. (2018) used an adapted oddball task with pure tones and found that ASD adults are less influenced by contextual manipulations (Goris et al. 2018). Other studies show different aMMN patterns for speech/phoneme versus pure‐tone stimuli compared with controls, despite similar overall amplitudes and latencies (Grisoni et al. 2019; Kasai et al. 2005). Regarding vMMN, only two studies have examined adult populations with ASD. Both found reduced vMMN amplitudes in ASD compared to neurotypical controls—one using facial stimuli (Kovarski et al. 2021) and the other using simple shapes (Cléry et al. 2013).

Within the broader autism spectrum, studies have also examined auditory and visual MMN responses in neurotypical individuals with varying levels of autistic traits (Mazer, Garcez, et al. 2024). This dimensional approach offers several advantages: It aligns with recent findings suggesting distinct genetic and neural profiles for social versus nonsocial autistic characteristics (Bralten et al. 2018; Warrier et al. 2019), allows for larger sample sizes, and helps reduce confounding factors often present in clinical populations, such as pharmaceutical interventions (Constantino and Todd 2003). However, the limited number of studies exploring either auditory or visual MMN in relation to autistic traits has not yielded consistent evidence (Mazer, Garcez, et al. 2024). Some studies have reported significant, yet conflicting, associations. For example, higher scores on the Autism‐Spectrum Quotient (AQ; Baron‐Cohen et al. 2001) have been linked to reduced aMMN (Zhao et al. 2015; Fan and Cheng 2014) and both increased (Ford et al. 2022) and decreased (Gayle et al. 2012) vMMN. Notably, the effect in the auditory domain appeared to be specific to traits related to communication and social difficulties (Zhao et al. 2015), while Ford et al. (2022) found effects exclusively in the communication and attention to detail subscales of the AQ, suggesting that MMN alterations may depend on both the sensory modality and the specific autistic trait dimension involved. Interestingly, all three studies employed social stimuli, namely, speech (Zhao et al. 2015) or faces (Ford et al. 2022; Gayle et al. 2012), leaving a gap in the literature regarding MMN responses elicited by nonsocial stimuli.

However, given the inconsistencies across studies and the limited number of investigations adopting a dimensional approach, that is, examining autistic traits or symptom severity as continuous variables within community and clinical samples rather than comparing categorical ASD vs. neurotypical groups, it remains difficult to draw robust conclusions about MMN modulation in ASD. Such an approach is theoretically grounded in models like the Hierarchical Taxonomy of Psychopathology (HiTOP), which conceptualize psychopathology as continuously distributed across the population (Ruggero et al. 2019). It is also empirically advantageous because it increases sample variability, minimizes clinical confounds (e.g., comorbidities), and allows for testing predictions from the PPF across the full trait spectrum, including emerging evidence for a dissociation between social and nonsocial autistic domains (Warrier et al. 2019).

In contrast to the mixed findings in ASD, the reductions in aMMN in SSD are now one of the more commonly observed neurophysiological correlates and are thought to be a biomarker of psychotic disorders (Donaldson et al. 2020, 2023). In the visual domain, the same effect is observed, albeit with a substantially smaller number of studies and a smaller effect size (Mazer, Carneiro, et al. 2024). MMN abnormalities have also been observed among high‐risk individuals, although these alterations tend to be less pronounced than those found in schizophrenia samples (Erickson et al. 2016). However, how such MMN deviations manifest across the broader nonclinical spectrum, specifically in individuals with greater schizotypal traits, remains less clear. To date, studies have reported only trend‐level associations, with no consistent evidence of robust effects (e.g., Broyd et al. 2016; J. M. Ford 2018; Ford et al. 2022). This lack of robust findings, combined with the modality‐specific inconsistencies observed in ASD research, underscores the need for studies that adopt a dimensional approach and explore MMN responses across both auditory and visual modalities. In addition, manipulating task difficulty offers a way to directly probe predictive processing mechanisms: When the perceptual distinction between standard and deviant stimuli becomes more subtle, the system may rely more heavily on prior expectations, generated by frequent standard stimuli, thereby reducing the need for a substantial update of the internal model (Kimura and Takeda 2013). Within the PPF, this makes trait‐related differences more likely to emerge: individuals with ASD, who underweight priors (van de Cruys et al. 2014), may show disproportionately larger MMN responses under difficult conditions, whereas those with SSD, who overweight priors (Sterzer et al. 2018; Corlett et al. 2019), may show diminished responses. In contrast, when deviations are large and easily discriminable, performance may approach the ceiling (Horváth et al. 2008), obscuring differences in predictive processing. Thus, task difficulty provides a principled experimental lever to test PPF‐related abnormalities in both under‐reliance and overreliance on priors.

Considering the PPF for the MMN component and the autistic‐positive schizotypy axis, we hypothesize that (H1) the MMN amplitude will be generally reduced in the difficult condition compared to the easy condition; (H2) autistic traits, particularly those related to restricted interest and detail orientation (RIDO), will be associated with larger MMN amplitudes; (H3) schizotypal traits, specifically positive symptoms, will be associated with smaller MMN amplitudes. We also expect that H2 and H3 will reveal larger effect sizes for the difficult task, considering that less perceptual distinction between standard and deviant stimuli could lead to increased reliance on priors, making trait differences in underweighting versus overweighting of priors more likely to emerge, whereas in the easy task, ceiling effects may obscure such trait‐related abnormalities. However, as evidence from nonclinical populations remains limited and mixed, these hypotheses should be considered tentative and primarily guided by theoretical predictions derived from the PPF.

## 2 Methods

The study protocol was publicly registered in the Open Science Framework prior to the data collection (https://osf.io/7t2kd) on April 23, 2023 (Mazer et al. 2023). The data used in this study were collected from the same cohort of participants as in a prior work from our group (Mazer et al. 2025), which investigated target trials and the P3b component. In the current analysis, the focus shifts to the MMN component as a computation of target minus standard stimuli, enabling the investigation of automatic deviant detection. The experimental protocol and participant details remain consistent with the earlier work; however, the data processing, analytical approach, and scientific objectives differ significantly. The reuse of this dataset has been appropriately acknowledged and complies with ethical guidelines.

A favorable appraisal was granted by the local Ethical Committee (Faculty of Psychology and Educational Sciences, University of Porto; Ref. 2022/09‐06) and by the Data Protection Office of the University of Porto (Ref. A‐T15/2023). All participants provided written informed consent before beginning the study and received a 5€ voucher compensation at the end of the experiment. Participant recruitment and data collection procedures for this study have been described previously (Mazer et al. 2025).

### 2.1 Participants

A community sample of 122 participants (62 female) between 18 and 60 years old (*M* = 26.99; SD = 8.97) was recruited online through e‐mail and other communication channels. This sample size was estimated in order to allow detecting an effect size of at least *f* ^*2*^ = 0.15 with 0.95 statistical power (details regarding the power analysis can be found in the study preregistration, https://osf.io/7t2kd (Mazer et al. 2023). All participants had fluency in Portuguese and normal or corrected‐to‐normal vision. Initial screening excluded participants if they reported a neurological diagnosis, substance abuse, or uncorrected motor or sensory deficits. Participants excluded during this screening did not proceed to the experimental phase. Due to the known comorbidities of those with high schizotypy and autistic traits, we did not exclude participants if they had any self‐reported psychiatric diagnoses to ensure the study was more generalizable to the population being studied. A total of eight participants reported a past history of psychiatric illness, namely, depression (*n* = 6), psychotic depression (*n* = 1), and anorexia (*n* = 1). In addition, 12 participants reported a current history of psychiatric illness, namely, anxiety (*n* = 8), attention‐deficit/hyperactivity disorder (ADHD) (*n* = 3), and comorbid ASD/ADHD with anxiety (*n* = 1).

Of the participants who reported a history of current or past psychiatric illness, seven reported medication intake at the time of the data collection, including antidepressants (*n* = 5), benzodiazepines (*n* = 1), and methylphenidate (*n* = 1). Additionally, three participants did not report being diagnosed with any psychiatric disorder but were taking antidepressants. Finally, seven participants reported a family history of ASD, and five reported a family history of SSD.

Following the initial screening process, additional exclusion criteria were applied during the ERP data analysis. Participants were excluded from the analysis for each task and modality if they met any of the following criteria: (1) presented errors in EEG acquisition (e.g., recording interruptions due to amplifier or software crash, missing event markers), (2) had less than 20 artifact‐free trials after segment rejection, (3) had more than 3 interpolated channels within the electrode cluster, and (4) were identified as outliers (*Z*‐scores of ± 3). Details on the number of participants excluded in each step can be found in the Supporting Information (Table S1).

### 2.2 Self‐Report Measures

A sociodemographic interview and a general health questionnaire were initially conducted to confirm inclusion criteria and gather information on important moderators. The sociodemographic data encompassed age, sex, handedness, education level, and musical training. The general health assessment included inquiries about sleep patterns, alcohol and drug use, neurological and psychiatric diagnoses, family history of clinical ASD or SSD, and current medications.

#### 2.2.1 Autistic Traits

Autistic traits were measured through the AQ, a self‐report instrument developed by Baron‐Cohen et al. (2001) to measure autism traits in adults of the general population. The AQ consists of 50 items and assesses autistic traits in five original factors (social skill, attention switching, attention to detail, communication, and imagination) using a 4‐point Likert scale (ranging from *definitely agree* to *definitely disagree*). Higher scores indicate higher autistic traits, and the scale scores range from 1 to 200. We considered the Portuguese adaptation of the scale (Barros et al. 2022), which reported a three‐factorial solution: social skills (*α* = 0.86), communication/imagination (*α* = 0.55), and RIDO (*α* = 0.57). Higher scores in the social skills dimension indicate deficits in social abilities, while higher scores in the communication dimension reflect difficulties with pragmatic language use. Similarly, higher scores in the RIDO dimension suggest a greater tendency to focus on details (Barros et al. 2022).

#### 2.2.2 Schizotypal Traits

Schizotypal traits were assessed through the Schizotypal Personality Questionnaire (SPQ; Raine 1991). SPQ is a 74‐item questionnaire with a dichotomous response format (Yes/No) and scores on nine subscales; four of positive schizotypy or cognitive–perceptual features (ideas of reference; odd beliefs/magical thinking, suspiciousness/paranoid ideation, and unusual perceptual experiences); four of negative schizotypy or interpersonal features (social anxiety, no close friends, suspiciousness/paranoid ideation, and constricted affect); and two of disorganized features (odd behavior and odd speech). Scoring was assigned as 1 for yes and 0 for no. The total score ranges from 0 to 74, with higher scores indicating greater schizotypal traits. For this study, we used the Portuguese‐validated version of the SPQ (Santos and Paixão 2013), which has a three‐factorial structure: positive schizotypy (Cronbach's *α* = 0.87), negative schizotypy (*α* = 0.88), and disorganized features (*α* = 0.78).

### 2.3 Experimental Tasks

**FIGURE 1:**
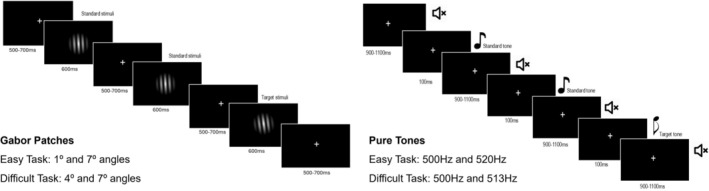
Schematic illustration of the experimental paradigms. In the visual paradigm, Gabor patches were presented for 600 ms and followed by a randomized interstimulus interval displaying a fixation cross for 500–700 ms. In the auditory paradigm, 100‐ms pure tones were presented and followed by a randomized interstimulus interval of 900–1100 ms.

The experimental tasks employed have been detailed previously (Mazer et al. 2025). The study included one auditory and one visual oddball task. Participants were seated at about 112 cm from the computer screen displaying the stimuli in an electrically shielded room. The tasks were designed and delivered using E‐Prime 2.0 software (2002) (Psychology Software Tools, Pittsburgh, PA). Each task modality included one training block and 4 experimental blocks (two easy and two difficult). The initial training block included a sequence of 5 stimuli (4 standards and 1 target). Each experimental block consisted of 152 stimuli (38 targets and 114 standards) per modality, meaning that target stimuli were presented with a 25% probability (see Figure 1 for a schematic representation of the tasks). Block order (easy vs. difficult; auditory vs. visual) was fully counterbalanced across participants, and within each block, the designation of target versus standard stimuli was alternated to control for stimulus‐specific effects (e.g., in one easy visual block, the 1° angle served as the target, and in the other block, the 7° angle did). The overall trial duration was about 1100–1300 ms per stimulus in both modalities. Participants were informed before each block which stimulus would serve as the target for that block and were also told that this target stimulus would always be the less frequent one in the sequence. Each participant was also instructed to press a button when they saw or heard the target stimulus. Because participants were instructed to attend to the target stimuli and provide a behavioral response, this design constitutes an active oddball paradigm. As such, the elicited MMN may include contributions from attentional processes in addition to the canonical automatic MMN response (see Fitzgerald and Todd 2020), a point we address in the Discussion and Limitations.

#### 2.3.1 Auditory Task

Stimuli consisted of two pure tones with different frequencies, each lasting 100 ms, presented at 90 dB through headphones. A fixation cross was displayed continuously during the auditory stimuli blocks, and participants were instructed to keep their eyes open and focused on the cross. The stimulus onset asynchrony was randomized between 1000 and 1200 ms. For the easy task, the stimulus frequencies were 500 and 520 Hz, and for the difficult task, the frequencies were 500 and 513 Hz. These frequencies were chosen based on results from a pilot experiment. The results of this pilot experiment can be found in the preregistered protocol https://osf.io/7t2kd (Mazer et al. 2023).

#### 2.3.2 Visual Task

Stimuli consisted of oblique Gabor patches on a black background, generated using an online patch generator (https://www.cogsci.nl/gabor‐generator). The parameters were defined for the following standard features: size: 5000 pixels; envelope: linear; spatial frequency: 0.02 cycles/pixel; phase: 0 cycles; background color: red 0, green 0, blue 0; color 1: red 255, green 255, blue 255; color 2: red 0, green 0, blue 0. The orientation of the gratings was manipulated to reach two levels of difficulty. For the easy task, the Gabor patches were shown with 1° and 7° angles, while for the difficult task, the angles were 4° and 7°. These angle variations were selected based on results from a pilot behavioral study detailed in the preregistered protocol. The Gabor patches were displayed for 600 ms, followed by a fixation cross. The fixation cross‐interval was randomized between 500 and 700 ms. Visual stimuli were presented on a 17‐in. screen with a 60‐Hz refresh rate. The Gabor patches were elliptical, measuring 13 cm vertically and 12 cm horizontally, corresponding to visual angles of approximately 6.52° vertically and 6.03° horizontally from a viewing distance of 112 cm (Figure 1).

### 2.4 EEG Acquisition and Processing

The EEG was recorded using a NetAmps 300 system from Electrical Geodesics Inc. (Eugene, Oregon, USA) with a 128‐channel hydrocel geodesic sensor net from the same company. The signal was digitized at 1000 Hz with an online vertex reference (Cz). The electrode impedances were kept below 50KΩ (high‐impedance amplifier). Before processing, the data were downsampled to 500 Hz.

Data processing and analysis were conducted using the EEGLAB toolbox (v2021.0) for MATLAB R2021a (2021) (Delorme and Makeig 2004;). Although not originally planned, data preprocessing was performed with the Harvard Automated Processing Pipeline, an automated preprocessing pipeline for EEG data (HAPPE+ER, Version 4.0, Monachino et al. 2022). First, CleanLine (Mullen 2012) was used to filter 50‐Hz line noise. Then, high‐pass and low‐pass finite impulse response (FIR) filters of 0.1 and 30 Hz were applied to the data. Artifact correction was performed using the wavelet thresholding approach with a hard margin to remove artifact data and isolate neural data. The continuous data were then segmented into epochs of −200–800 ms relative to stimulus onset and were baseline corrected (−200–0 ms). Following this, bad channel identification and interpolation within the epochs were conducted. Conducting bad channel identification within segments allows the user to minimize the number of epochs removed during trial rejection, while maximizing the correction of artifacts within each segment (Monachino et al. 2022). Electrodes were deemed noisy and rejected if their joint probability exceeded the mean of the activity of all other electrodes by 3 SD. Finally, automatic trial rejection was implemented by using both the segment amplitude and similarity criteria. Thresholds for the minimum and maximum amplitude were set at −150 and 150 mV in line with HAPPE recommendations (Gabard‐Durnam et al. 2018). Preprocessing HAPPE parameters are available in Table S2. The sensitivity of the HAPPE procedures allows for more trials to be kept. In addition, HAPPE ensures a more consistent and reproducible approach to data processing, minimizing human error and variability inherent in manual steps, enhancing the reliability of the ERP analysis, and offering quantitative measures of data quality (Hudac and Webb 2024). Outputs regarding data quality and pipeline performance can also be found in the Supporting Information.

A table with the mean (*M*) and standard deviation (SD) number of correct trials postartifact rejection can be found in the Supporting Information (Table S3). Additionally, correlation analysis revealed no significant correlations between the number of included trials in the EEG analysis (postartifact rejection) and the self‐report measures (*p*s > 0.05, Table S4).

### 2.5 ERP Measures

The mean amplitude of the MMN ERP component was computed as target minus standard stimuli over time windows selected based on established literature and a visual inspection of the ERP plots. For the aMMN, a time window of 150–300 ms was used in the Fz electrode cluster (Luck 2014; Näätänen et al. 2004). For the vMMN, a time window of 150–350 ms was used in the Pz electrode cluster (Kimura et al. 2010; Stefanics et al. 2014). The preregistered time window (100–250 ms) was adjusted after inspecting the grand‐average plots, which showed a latency delay shift, likely related to task difficulty.

For this high‐density EEG setup, channels of interest were defined as the regional average of locations defined by the extended international 10–5 system. Fz was the average of electrodes E4, E10, E11, E16, E18, and E19, and Pz was the average of electrodes E61, E62, E67, E72, E77, and E78. EEG cluster data were eliminated from further analysis if more than three electrodes from each cluster were derived from interpolated channels (criterion 3 for exclusion, Table S1). This parameter was considered to avoid signal distortion, as excessive interpolation can compromise the spatial precision of ERP signals. This approach replaces the preregistered plan, which specified that filtered data would be visually inspected to reject drifting or flat‐lined channels, with a limit of excluding no more than 10% of electrodes per recording. Instead, data were processed using the HAPPE+ER pipeline, Version 4.0 (Monachino et al. 2022), which integrates automated artifact handling procedures in place of manual exclusions.

### 2.6 Statistical Analysis

All analyses were performed using IBM SPSS software version 29.0.2.0. Results were expressed as mean ± standard deviation. Separate paired sample *t*‐tests with Bonferroni correction were conducted for visual and auditory MMN amplitudes in the easy and difficult conditions.

General linear models, specifically independent multivariate linear regressions for each MMN component, were applied to test the extent to which dimensions of the schizotypal and autistic traits converge with the computed ERP for each task difficulty condition and stimulus modality. Model 1 included, at the same time, the three dimensions of AQ scores (social skills, communication/imagination, and RIDO) as predictors of MMN amplitudes in the task condition (easy and difficult) and modality (visual and auditory). Model 2 includes the three dimensions of SPQ (Positive, Negative, and Disorganized Features) as predictors of MMN amplitudes in the task condition (easy and difficult) and modality (visual and auditory).

To assess the strength of evidence and obtain more conservative results, we conducted Bayesian hypothesis testing. Nonsignificant effects from Models 1 and 2 were re‐analyzed as simple Bayesian linear regressions in JASP (JASP Team 2024) using a default prior r scale = 0.354. Following guidelines for categorizing Bayes Factors (BF_10_, van Doorn et al. 2021), the resulting outcomes can be quantified as follows: *BF_10_* of 1 = equal support for the null and the alternative hypothesis, 1–3 = weak evidence in favor of the alternative hypothesis, and 3–10 = moderate evidence in favor of the alternative hypothesis. Conversely, to quantify evidence for the null, *BF_10_* between 0.33 and 1 represents weak evidence for the null, while *BF_10_* between 0.10 and 0.33 represents moderate evidence for the null.

## 3 Results

### 3.1 Task

Full task manipulation behavioral results are presented in a previous publication (Mazer et al. 2025). Participants showed a clear effect of increased task difficulty across both modalities. In the difficult condition, targets elicited significantly slower responses (auditory: *M* = 503 ms; visual: *M* = 496 ms) compared to the easy condition (auditory: *M* = 461 ms; visual: *M* = 434 ms), *p*s < 0.001, *d*s = 0.63–1.56. Accuracy was likewise reduced in the difficult condition (auditory: 73%; visual: 76%) relative to the easy condition (auditory: 85%; visual: 94%), *p*s < 0.001, *ds* = 0.72–1.15. The behavioral results provide support for the effectiveness of the task difficulty manipulation, with increased difficulty reflected in slower responses and reduced accuracy.

**FIGURE 2:**
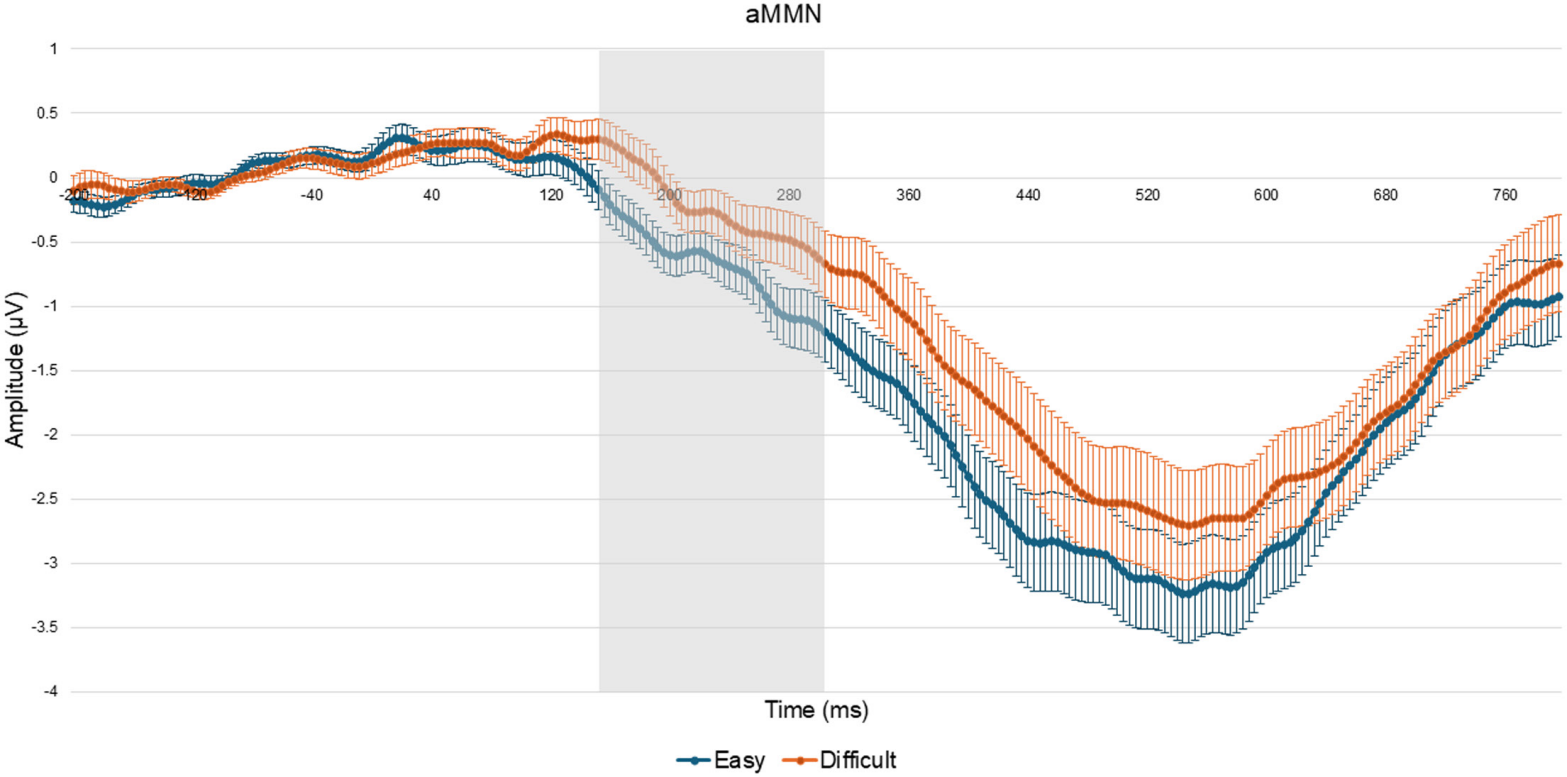
Grand average target minus standard waveform at the Fz electrode cluster for the auditory easy and difficult trials. Error bars indicate ± 1 standard error of the mean (SEM). The gray area represents the 150‐ to 300‐ms time window corresponding to the auditory MMN.

**FIGURE 3:**
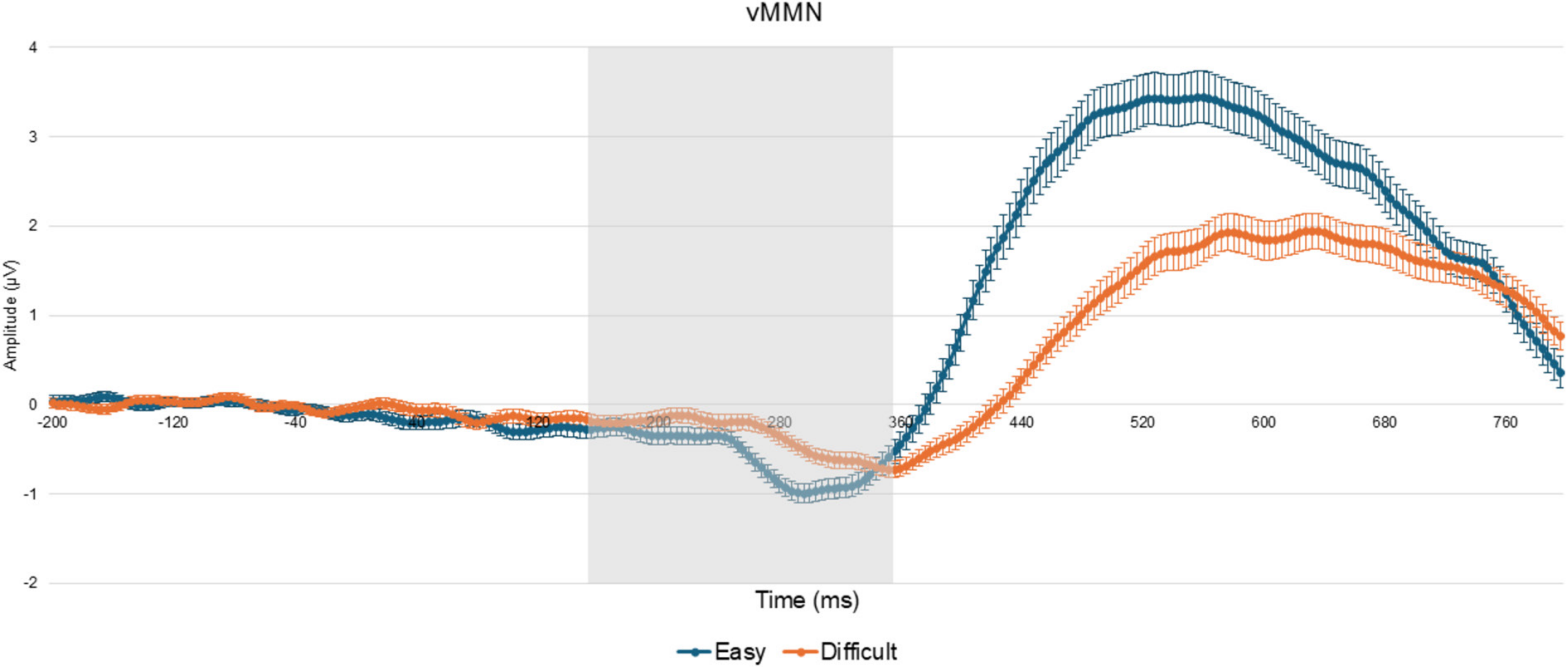
Grand average target minus standard waveform at the Pz electrode cluster for the visual easy and difficult trials. Error bars indicate ± 1 standard error of the mean (SEM). The gray area represents the 150‐ to 350‐ms time window corresponding to the visual MMN.

**FIGURE 4:**
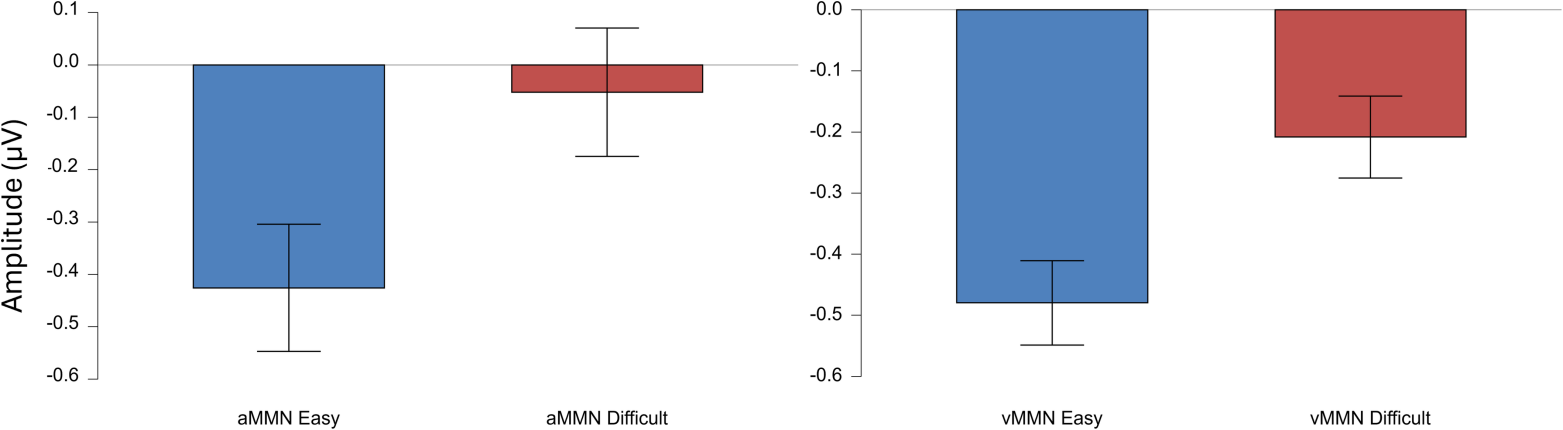
Mean auditory (aMMN) and visual (vMMN) mismatch negativity amplitudes across task difficulty tasks. Error bars represent standard deviations of the mean (SD).

A paired sample *t*‐test revealed significant differences between tasks for both the auditory (*t*(107) = −2.095, *p* = 0.019, *d* = 1.826, Figure 2) and visual (*t*(106) = −3.270, *p* < 0.001, *d* = 1.005, Figure 3) modalities. For the auditory modality, MMN amplitude was larger in the easy (*M* = −0.426, SD = 1.391) compared to the difficult task (*M* = −0.052, SD = 1.511). Similarly, in the visual modality, MMN amplitude was greater in the easy (*M* = −0.480, SD = 0.772) compared to the difficult task (*M* = −0.208, SD = 0.756, Figure 4). Both effects remained statistically significant after Bonferroni correction (*p* < 0.025, Table 1).

**TABLE 1:** Means and standard deviations of auditory and visual MMN amplitudes for each condition.

	*N*	Mean	*SD*	Minimum	Maximum
aMMN easy	110	−0.426	1.391	−4.788	3.153
aMMN difficult	109	−0.052	1.511	−4.574	5.217
vMMN easy	106	−0.480	0.772	−2.532	2.483
vMMN difficult	109	−0.208	0.756	−2.320	1.491

Abbreviations: a, auditory; SD, standard deviation; v, visual.

### 3.2 Autistic and Schizotypal Traits

The SPQ dimensions were all moderately correlated with each other (*r*‐values from 0.344 to 0.485, all *p*s < 0.001, Table S4). Regarding AQ, only communication difficulties and social skills deficits were positively correlated (*r*(120) = 0.207, *p* = 0.022). AQ RIDO did not correlate with either communication (*r*(120) = −0.133, *p* = 0.145) or the social skills dimensions (*r*(120) = 0.045, *p* = 0.621). Across scales, the AQ RIDO correlated with the SPQ Disorganized (*r*(120) = 0.252, *p* = 0.005), while the negative schizotypy covaried with AQ deficits in social skills (*r*(120) = 0.408, *p* < 0.001). No other statistically significant correlations were found (all *p*s > 0.092, Table S4).

Higher AQ RIDO scores were associated with larger vMMN amplitudes exclusively in the difficult task (*AdjR* ^*2*^ = 0.044, *F*(1,90) = 4.149, *B* = −0.045, *p* = 0.045, *BF_10_* = 0.39). In the opposite direction, higher AQ communication scores were associated with smaller aMMN amplitudes in the easy task (*AdjR* ^*2*^ = 0.049, *F*(1,90) = 4.647, *B* = 0.111, *p* = 0.034, *BF_10_* = 0.35). However, after applying the Bonferroni correction, these results were no longer significant (*p* > 0.0125), and the Bayes factor provides moderate evidence in favor of the null. No significant associations were found for either aMMN (*p* > 0.358) or vMMN (*p* > 0.246; Table 2). Most corresponding Bayes factors (*BF_10_* < 0.52) provided at least weak to moderate evidence in favor of the null hypothesis. The exception was the association between AQ social skills and vMMN in the easy task, which yielded inconclusive evidence (*BF_10_* = 1.08).

**TABLE 2:** Regression analysis of Autism‐Spectrum Quotient (AQ) dimensions on auditory (aMMN) and visual (vMMN) amplitudes for the easy and difficult (diff) tasks.

Variable	Parameter	*B*	SE	*t*	*p*	% CI	*BF*_*10*_
aMMN easy	AQ S. skills	−0.023	0.025	−0.923	0.358	[−0.073, 0.027]	0.24
	**AQ com**.	**0.111**	**0.052**	**2.156**	**0.** **034**	**[0.009, 0.214]**	0.35
	AQ RIDO	0.033	0.043	0.773	0.442	[−0.052, 0.118]	0.32
aMMN diff.	AQ S. skills	0.002	0.027	0.077	0.939	[−0.052, 0.056]	0.22
	AQ com.	0.037	0.056	0.657	0.513	[−0.074, 0.148]	0.22
	AQ RIDO	0.010	0.046	0.222	0.825	[−0.082, 0.102]	0.20
vMMN easy	AQ S. skills	0.018	0.014	1.264	0.209	[−0.010, 0.047]	1.08
	AQ com.	0.028	0.030	0.940	0.350	[−0.031, 0.087]	0.43
	AQ RIDO	−0.020	0.025	−0.819	0.415	[−0.069, 0.026]	0.28
vMMN diff.	AQ S. skills	0.015	0.013	1.169	0.246	[−0.011, 0.041]	0.52
	AQ com.	0.028	0.027	1.047	0.298	[−0.081, 0.025]	0.22
	**AQ RIDO**	**−0.045**	**0.022**	**−2.037**	**0.** **045**	**[−0.088, −0.001]**	0.39

*Note:* Significant coefficients (*p* < .05) are shown in bold.

Abbreviations: a, auditory; com., communication; S. skills, social skills; v, visual.

Regarding schizotypy subdimensions, no significant associations were found for either aMMN (*p* > 0.080) or vMMN (*p* > 0.135; Table 3). The corresponding Bayes factors generally indicated weak to moderate evidence in favor of the null hypothesis (*BF_10_* < 0.86). The exception was the association between SPQ Disorganized traits and vMMN in the difficult task, which yielded inconclusive evidence with roughly equal support for the presence or absence of an effect (*BF_10_* = 1.09).

**TABLE 3:** Regression analysis of schizotypal personality questionnaire (SPQ) dimensions on auditory (aMMN) and visual (vMMN) amplitudes for the easy and difficult (diff) tasks.

Variable	Parameter	*B*	SE	*t*	*p*	% CI	*BF*_*10*_
aMMN easy	SPQ disor.	0.071	0.051	1.385	0.169	[−0.031, 0.172]	0.45
	SPQ positive	−0.049	0.028	−1.771	0.080	[−0.104, 0.006]	0.27
	SPQ negative	0.031	0.024	1.259	0.211	[−0.018, 0.079]	0.25
aMMN diff.	SPQ disor.	0.078	0.051	1.425	0.158	[−0.031, 0.186]	0.39
	SPQ positive	−0.023	0.028	−0.784	0.435	[−0.082, 0.035]	0.86
	SPQ negative	0.013	0.024	0.501	0.618	[−0.039, 0.065]	0.21
vMMN easy	SPQ disor.	−0.014	0.030	−0.464	0.644	[−0.073, 0.045]	0.22
	SPQ positive	0.018	0.016	1.140	0.257	[−0.014, 0.050]	0.20
	SPQ negative	−0.006	0.014	−0.388	0.699	[−0.034, 0.023]	0.25
vMMN diff.	SPQ disor.	0.040	0.027	1.506	0.135	[−0.013, 0.093]	1.09
	SPQ positive	0.004	0.014	0.292	0.771	[−0.024, 0.033]	0.50
	SPQ negative	−0.011	0.013	−0.894	0.374	[−0.036, 0.014]	0.21

Abbreviations: a, auditory; disor., disorganized; v, visual.

We additionally conducted a unified regression analysis that included all AQ and SPQ subscales simultaneously, as some dimensions may not operate as fully independent, as suggested by the weak‐to‐moderate correlations observed across scales. These exploratory results are reported in the Supporting Information (Table S5) and did not materially change our conclusions.

## 4 Discussion

In this study, our aim was to (1) investigate whether the auditory and visual MMN amplitudes were influenced by the task difficulty and (2) understand how these components may vary across autistic and schizotypal trait dimensions.

### 4.1 Task

We found that increased task difficulty led to a significant reduction in MMN amplitude for both the auditory and visual modalities, confirming our first hypothesis (H1). To our knowledge, only two previous studies have directly examined both sensory domains within the same sample while applying difficulty manipulations across modalities (Alho et al. 1992; Woods et al. 1992), and both reported increased aMMN and vMMN with higher difficulty, although each was conducted with relatively small samples of 14 and 12 young adults, respectively. In the auditory domain more broadly, findings have been mixed: Some studies report no modulation of MMN amplitude under increased perceptual load (see Ghani et al. 2020, for a review), whereas others, consistent with our results, have observed reduced aMMN amplitudes under higher task demands (Amenedo and Escera 2000; Campbell and Davalos 2015; Kramer et al. 1995; Pakarinen et al. 2007). In the visual domain, while the vMMN has been less extensively studied, our results align with previous findings on orientation discrimination, where a greater difference between stimuli is associated with enhanced vMMN amplitudes (Male 2025).

Under predictive processing assumptions, our findings of reduced MMN amplitudes with increased task difficulty align with the idea that in more challenging conditions, the perceptual distinction between standard and deviant stimuli becomes less pronounced. This reduced salience likely leads individuals to rely more on top–down expectations, resulting in smaller prediction errors and, consequently, diminished MMN responses. However, the mixed findings in the existing literature indicate that this relationship is not straightforward and may depend on specific task parameters or stimulus features.

It is important to note that our design employed an active oddball task, requiring participants to attend and respond to deviants. As highlighted by Fitzgerald and Todd (2020), such designs alter the canonical interpretation of MMN as a purely pre‐attentive marker, since attentional processes and overlapping components may contribute to the observed negativity. Thus, this consideration means that our results cannot be interpreted as reflecting pre‐attentive change detection alone. Instead, the reduced amplitudes observed under higher task difficulty likely reflect a combination of prediction error signaling and attentional resource allocation.

### 4.2 Autistic Traits

Our hypothesis (H2) suggested an increase of MMN with autistic traits, as suggested by the PPF—individuals with higher autistic traits seem to rely on previous information (i.e., priors) to a lower extent and more on immediate sensory inputs (Pellicano and Burr 2012).

Although we found trend‐level associations with the RIDO and communication domains of autistic traits, the corresponding Bayes factors ultimately revealed moderated evidence for an absence of an effect. Evidence from autism spectrum and MMN in both adult clinical (Vassall et al. 2025) and adult nonclinical samples (Mazer, Garcez, et al. 2024) is far from being consistent and homogeneous. Interestingly, even at a clinical level, although individual studies have reported differences in the aMMN (Fan and Cheng 2014; Goris et al. 2018; Kujala et al. 2005, 2007) and vMMN (Cléry et al. 2013), a meta‐analysis (Vassall et al. 2025) suggests no significant group differences between autistic and nonautistic individuals in the auditory or visual oddball neural signature of deviation detection. Vassall et al. (2025), in their meta‐analysis, acknowledge that sensory processing differences in autism may become more pronounced as stimulus complexity increases, such as in tasks involving visual discrimination of speech or faces. In contrast, our study, which manipulated task difficulty using relatively simple stimuli (pure tones and Gabor patches), did not reveal significant differences related to autistic traits. This may reflect the limited complexity of the stimuli, which might not be sufficiently challenging to index predictive processing mechanisms in a way that reveals group or trait‐based differences.

A closer examination of studies with community samples further highlights the complexity of findings in this area. For the auditory modality, Zhao et al. (2015) reported reduced aMMN amplitudes associated with higher scores on the AQ communication and social skills subscales but found no significant correlations with (lack of) imagination, attention switching, or attention to detail. Similarly, Fan and Cheng (2014) examined total AQ scores in both neurotypical and ASD groups and observed that higher scores were linked to reduced aMMN amplitudes but only when both groups were combined. This suggests that larger samples or the inclusion of extreme values may be necessary to detect such effects. The vMMN literature is even more limited, as also noted by Vassall et al. (2025). In nonclinical samples, Gayle et al. (2012) reported reduced vMMN amplitudes, whereas Ford et al. (2022) found the opposite pattern, with increased amplitudes. Importantly, both studies used socially salient facial stimuli, which may engage different processes than the nonsocial Gabor patches used in our paradigm. Moreover, while Gayle et al. (2012) restricted their analyses to total AQ scores, Ford et al. (2022) extended the approach to subscales and reported effects specifically for communication difficulties and attention to detail.

Thus, our findings, although only at trend‐level, align partly with this literature. Consistent with Zhao et al. (2015), we found reduced aMMN amplitudes linked to higher communication difficulties, but only in the auditory modality. In contrast, and in line with Ford et al. (2022), we observed a trend‐level increase in vMMN amplitudes associated with RIDO, a subscale specifically adapted for the Portuguese population (Barros et al. 2022). Together, these results suggest that social dimensions of autistic traits are more likely to modulate auditory MMN, whereas nonsocial, detail‐oriented traits appear more consistently linked to visual MMN alterations. Importantly, AQ RIDO did not correlate with either the communication or social skills dimensions, suggesting that these traits may tap, indeed, into distinct cognitive mechanisms. This emerging divergence is consistent with growing evidence for separate neural and genetic foundations underlying social and nonsocial features of autism (Bralten et al. 2018; Warrier et al. 2019).

Beyond modality, our study also showed that task difficulty modulated these patterns: Communication difficulties were associated with reduced aMMN only in the easy task, whereas RIDO scores predicted vMMN only in the difficult task. Our rationale for manipulating task difficulty was grounded in the PPF (see Introduction), where increasing difficulty was expected to yield more robust effects by reducing potential ceiling effects. This was indeed the case for the vMMN, which showed associations exclusively in the difficult condition. Such findings are consistent with the detail‐focused orientation reported in autism and may help explain superior visual performance often described in this population (Happé and Frith 2006; Cribb et al. 2016). In contrast, the easy visual task may have been insufficiently sensitive to capture these differences.

For the auditory modality, associations with communication difficulties emerged only in the easy task. Social and communication difficulties have been linked to auditory alterations (Shukla et al. 2020), which may account for this modality‐specific effect. Moreover, communication difficulties, as part of the social domain of autism, are not unique to the autism spectrum but also occur in conditions such as depression and anxiety (Kupferberg and Hasler 2023; Lacombe et al. 2024). This suggests that the expected pattern of stronger associations under higher difficulty might be more specific to the nonsocial domain of ASD, which is less likely to overlap with other psychopathological spectra. Although we anticipated reduced aMMN amplitudes in the difficult condition as well, the overall decrease in auditory MMN with higher task demands may have reduced statistical power to detect significant associations. Notably, the observed correlation in the difficult condition, although not statistically significant, was in the same direction as the significant effect found in the easy task.

Hence, although we did not find statistically significant associations between MMN and autistic traits, our study contributes valuable evidence to the ongoing search for neurobiological mechanisms underlying the autism spectrum. This contribution is particularly important given the current lack of studies focused on adult populations and research examining MMN in the visual domain—areas that remain underrepresented in the existing literature.

### 4.3 Schizotypal Traits

Our findings regarding schizotypal traits were unexpected, particularly given the consistent evidence of reduced MMN amplitudes in individuals with SSDs and those at clinical high risk (Donaldson et al. 2023; Erickson et al. 2016; Mazer, Carneiro, et al. 2024). Based on this robust clinical literature, we anticipated observing a similar pattern for positive schizotypy in the general population (H3), in line with the view that schizotypal traits lie on a continuum with clinical psychosis (Raine 1991). Contrary to this expectation, we found no significant associations between MMN amplitude and schizotypal traits, with Bayesian analyses providing moderate evidence in favor of the null for both the positive and negative domains.

Nonetheless, these findings align to some degree with prior studies in nonclinical populations, which have similarly reported weak or inconsistent associations. For example, Ford et al. (2022), using a vMMN face‐paradigm, observed a trend toward increased vMMN amplitude with higher positive schizotypy—opposite to what is typically found in clinical SSD—although this effect did not survive Bonferroni correction. In the auditory domain, Erickson et al. (2024) found reduced MMN in patients with schizophrenia but no differences between nonclinical participants with and without voice‐hearing experiences.

Taken together, this suggests that MMN, particularly in nonclinical populations, may not reliably capture prediction error signals directly linked to the manifestation of positive symptoms, such as hallucinations. While aMMN reduction in SSD is among the most robust neurophysiological markers of the disorder and has even been proposed as a trait biomarker for psychosis spectrum disorder (Donaldson et al. 2023), this effect does not appear to extend clearly to subclinical or trait‐level expressions of schizotypy. These findings highlight a potential limitation of MMN as a generalizable marker of positive symptoms across the full psychosis spectrum (Andersen 2022) and underscore the need for further research to clarify the neural mechanisms underlying predictive processing in nonclinical schizotypy.

### 4.4 Limitations and Future Studies

While this research reports informative results with innovative methodology, some methodological and sample‐related limitations should be taken into consideration. First, our paradigm was active (participants attended/responded to deviants) rather than passive, which likely increased attentional influences on MMN estimates. Second, the number of deviant trials per condition (76) was lower than typical recommendations for maximal reliability, potentially reducing the signal‐to‐noise ratio. Third, we did not include an adaptation control (e.g., equiprobable/cascadic), limiting our ability to fully disentangle prediction error‐related activity from adaptation. Fourth, although we opted for a transparent and automated preprocessing pipeline (HAPPE+ER), this approach is still relatively new compared with traditional ICA/manual workflows and should be interpreted in that context. Fifth, despite including an initial pilot behavior study and harmonizing overall trial timing and stimulus/design differences across modalities (pure tones vs. Gabor patches; modality‐specific time windows and regions of interest [ROIs]) as well as the inherent differences in the basic processing mechanisms, we limit direct cross‐modal comparability.

In addition, sample‐related factors may have affected sensitivity: one limitation of our analyses concerns the inclusion of participants who self‐reported psychiatric conditions and/or medication use (*n* = 20). While consistent with dimensional frameworks, this may have introduced additional heterogeneity and unexplained variance in MMN measures. Given the small number of cases and the heterogeneity of medications and diagnoses reported, we did not consider these as covariates. Instead, we repeated the analyses excluding these participants, and the overall pattern of results remained unchanged, suggesting that medication use or history of psychiatric diagnosis was not the main determinants of our findings. Additionally, although we aimed to reach a broad community sample, our participants were primarily university students. This demographic homogeneity may not fully reflect the trait distribution in the general population, particularly in terms of cognitive and social functioning. This might also have contributed to the relatively low levels of positive schizotypy and the absence of clear phenotypical dissociations within schizotypal dimensions in our sample. Finally, analysis‐specific exclusions (e.g., outliers, excessive interpolation, or equipment failures) reduced the effective sample size (N) in some models, thereby lowering statistical power for small effects.

Future research should aim to determine whether the modulation of MMN by task difficulty observed in our study extends to other sensory modalities, such as the somatosensory domain. Additionally, there is a clear need to expand the evidence base for vMMN paradigms, which remain underrepresented compared to auditory studies. Future studies would benefit from including both simple and complex stimuli (e.g., social and emotional) within the same experimental design, allowing for a more nuanced understanding of how stimulus complexity interacts with autistic and schizotypal traits. It is also crucial to incorporate measures that differentiate between social and nonsocial autistic traits, as emerging evidence suggests these dimensions may relate to distinct neural processes. This multidimensional approach could provide a more comprehensive understanding of how individual differences shape prediction error signalling across modalities and contexts. As the present study did not include measures to disentangle magnitude of deviance from task difficulty, we interpret our findings primarily in terms of task difficulty, consistent with our preregistration. However, future studies should aim to isolate these two factors using designs and methodologies specifically developed to differentiate them.

### 4.5 Conclusion

This study investigated how task difficulty modulates auditory and visual MMN and how these neural markers relate to autistic and schizotypal traits in a large adult community sample. We found that increasing task difficulty reduced MMN amplitude across both modalities, consistent with predictive processing accounts that emphasize the role of perceptual load in shaping prediction error signals.

Although associations with schizotypal traits were not significant after correction, exploratory analyses revealed modality‐ and difficulty‐specific links with autistic traits: Communication difficulties were associated with smaller auditory MMN in the easy task, whereas RIDO were linked to larger visual MMN in the difficult task. This pattern suggests that distinct social and nonsocial autistic dimensions may differentially shape prediction error responses, particularly when perceptual demands increase.

Together, these findings underscore the value of examining both auditory and visual modalities within the same design, while also adopting a dimensional perspective on traits. It is important to note that our paradigm employed an active task to elicit MMN, which may have influenced attentional contributions to the response. Future research should build on this approach by testing more diverse samples and further probing how task manipulations interact with specific trait dimensions to influence predictive processing mechanisms.

## Author Contributions

**Prune Mazer:** conceptualization, formal analysis, funding acquisition, investigation, methodology, validation, writing – original draft. **Rita Pasion:** conceptualization, formal analysis, methodology, supervision, validation, writing – review and editing. **Zohra Kamran Rao:** visualization, writing – original draft. **Celeste Silveira:** supervision, writing – review and editing. **Fernando Ferreira‐Santos:** conceptualization, methodology, supervision, writing – review and editing.

## Funding

This work was supported by Fundação para a Ciência e a Tecnologia (FCT), under the grant reference 2021.07199.BD and DOI 10.54499/2021.07199.BD awarded to Prune Mazer. HEI‐Lab R&D Unit is funded by FCT (UID/05380/2025, UID/PRR/05380/2025, UID/PRR2/05380/2025). Further support was provided by project grants from FCT (2023.12283.PEX; ) and Cooperativa de Formação e Animação Cultural ‐ COFAC (FAZER+/ILIND/HEI‐Lab/1/2024) awarded to Rita Pasion.

## Conflicts of Interest

The authors declare no conflicts of interest.

## Supporting information




**Table S1:** Number of participants excluded per reason for exclusion. Participants were excluded from the ERP data analyses for each task if they met one of the following criteria: (1) presented errors in EEG acquisition, (2) had less than 20 artifact‐free trials after segment rejection, (3) had more than 3 electrodes from the cluster were interpolated, and (4) were identified as outliers (*Z*‐scores of ± 3). Conditions are categorized by task: auditory (A) or visual (V), trial type: standards (Std) or targets (Tag), and condition: easy (E) or difficult. (D).
**Table S2:** Parameters used in HAPPE processing.
**Table S3:** Descriptive statistics of EEG trials included postartifact rejection per participant. Each task (easy and difficult) included 76 targets and 228 standards before artifact rejection per participant.
**Table S4:** Correlations table between self‐report measures of the schizotypal personality questionnaire (SPQ) and the autism‐spectrum quotient (AQ) and EEG trials post artifact rejection and outliers removed. EEG conditions are categorized by task: auditory (A) or visual (V), trial type: standards (Std) or targets (Tag), and condition: easy (E) or difficult (D).
**Table S5:** Unified regression model including all AQ and SPQ subscales predicting auditory and visual MMN amplitudes across task difficulty conditions.

**Data S1:** Supporting Information.

**Data S2:** Supporting Information.

## Data Availability

The data that support the findings of this study and the task paradigms employed are openly available in the Open Science Framework (OSF) at https://osf.io/5sz7p/ (DOI: 10.17605/OSF.IO/5SZ7P).
