# Supplementary material for: Analysis of Task Demand Effects on Visual and Auditory Mismatch Negativity (MMN) Across Autistic and Schizotypal Traits
Source: Eur J Neurosci. 2026 Apr 10;63(7):e70490. doi: 10.1111/ejn.70490 (PMC13067925; doi:10.1111/ejn.70490)
Supplement: Supplementary file 1 — Table S1: Number of participants excluded per reason for exclusion. Participants were excluded from the ERP data analyses for each task if they met one of the following criteria: (1) presented errors in EEG acquisition, (2) had less than 20 artifact‐free trials after segment rejection, (3) had more than 3 electrodes from the cluster were interpolated, and (4) were identified as outliers (Z‐scores of ± 3). Conditions are categorized by task: auditory (A) or visual (V), trial type: standards (Std) or targets (Tag), and condition: easy (E) or difficult. (D). Table S2: Parameters used in HAPPE processing. Table S3: Descriptive statistics of EEG trials included postartifact rejection per participant. Each task (easy and difficult) included 76 targets and 228 standards before artifact rejection per participant. Table S4: Correlations table between self‐report measures of the schizotypal personality questionnaire (SPQ) and the autism‐spectrum quotient (AQ) and EEG trials post artifact rejection and outliers removed. EEG conditions are categorized by task: auditory (A) or visual (V), trial type: standards (Std) or targets (Tag), and condition: easy (E) or difficult (D). Table S5: Unified regression model including all AQ and SPQ subscales predicting auditory and visual MMN amplitudes across task difficulty conditions. [file EJN-63-0-s002.docx]

**Table S1.**

*Number of participants excluded per reason for exclusion. Participants were excluded from the ERP data analyses for each task if they met one of the following criteria: 1) presented errors in EEG acquisition, 2) had less than 20 artifact-free trials after segment rejection, 3) had more than 3 electrodes from the cluster were interpolated, and 4) were identified as outliers (Z-scores of ±3). Conditions are categorized by task – Auditory (A) or Visual (V) – trial type – Standards (Std) or Targets (Tag) – and condition – Easy (E) or Difficult. (D).*

| **Condition** | **1)** | **2)** | **3) Fz/Pz** | **4)** |
| --- | --- | --- | --- | --- |
| Easy Auditory | 2 | 0 | 8 | 2 |
| Easy Visual | 2 | 0 | 12 | 2 |
| Difficult Auditory | 3 | 0 | 8 | 2 |
| Difficult Visual | 1 | 0 | 12 | 0 |

**Table S2.**

*Parameters used in HAPPE processing.*

| Density | High (>30 channels) |
| --- | --- |
| Resting State or Task | Task |
| Acquisition Layout | 128 channel EGI HydroCel Geodesic Sensor Net |
| Channels | All |
| Line Noise Frequency | 50 Hz |
| Line Noise Reduction Method | CleanLine - Default |
| Resample | Off |
| Filter - Lowpass Cutoff | 30 Hz |
| Filter - Highpass Cutoff | 0.1 Hz |
| Filter Type | EEGLAB’s FIR |
| Bad Channel Detection | On, after wavelet thresholding |
| Bad Channel Detection Method | Default |
| Wavelet Thresholding | Default |
| Wavelet Threshold Rule | Hard |
| Segmentation | On |
| Segment start | -200 |
| Segment end | 800 |
| Baseline Correction | On, -200 to 0 |
| Interpolation within each segment | On |
| Segment Rejection Method | Both amplitude and similarity criteria |
| Minimum Segment Rejection Threshold | - 150 |
| Maximum Segment Rejection Threshold | 150 |
| Segment Rejection based on All Channels or ROI | All Channels |
| Flatline or all zero reference | Yes, Cz |
| Re-Reference Method | Average |

**Table S3.**

*Descriptive statistics of EEG trials included post-artifact rejection per participant. Each task (Easy and Difficult) included 76 targets and 228 standards before artifact rejection per participant.*

| **Condition** | **N** | **Min** | **Max** | **Mean** | ***SD*** |
| --- | --- | --- | --- | --- | --- |
| Trials A Std E | 119 | 72 | 200 | 147.08 | 20.80 |
| Trials V Std E | 121 | 70 | 187 | 141.21 | 21.64 |
| Trials A Std D | 118 | 74 | 184 | 148.53 | 18.63 |
| Trials V Std D | 121 | 89 | 208 | 146.02 | 22.10 |
| Trials A Tag E | 119 | 30 | 68 | 50.21 | 7.91 |
| Trials V Tag E | 120 | 23 | 67 | 48.53 | 8.37 |
| Trials A Tag D | 118 | 23 | 68 | 50.29 | 7.93 |
| Trials V Tag D | 121 | 28 | 67 | 50.07 | 7.87 |

*Note.* *SD* – Standard Deviation

**Table S4.**

*Correlations table between self-report measures of* *the Schizotypal Personality Questionnaire (SPQ) and the Autism-Spectrum Quotient (AQ) and EEG trials post artifact rejection and outliers removed. EEG conditions are categorized by task – Auditory (A) or Visual (V) – trial type – Standards (Std) or Targets (Tag) – and condition – Easy (E) or Difficult (D).*

| Variable | 1 | 2 | 3 | 4 | 5 | 6 |
| --- | --- | --- | --- | --- | --- | --- |
| 1. SPQ Disorganized | — |  |  |  |  |  |
| 2. SPQ Positive | .49^**^ | — |  |  |  |  |
| 3. SPQ Negative | .34^**^ | .48^**^ | — |  |  |  |
| 4. AQ Social Skills | .12 | .01 | .41^**^ | — |  |  |
| 5. AQ RIDO | .25^*^ | .14 | .11 | .05 | — |  |
| 6. AQ Communication | .10 | −.02 | .09 | .21 | −.13 | — |
| 7. Trials A Std E | .10 | −.04 | .05 | .13 | −.00 | .04 |
| 8. Trials V Std E | .03 | .14 | .10 | .07 | −.05 | −.08 |
| 9. Trials A Std D | −.09 | −.01 | .06 | .04 | .03 | .07 |
| 10. Trials V Std D | −.18 | −.02 | −.13 | −.04 | −.17 | .01 |
| 11. Trials A Tag E | .00 | −.00 | −.04 | .04 | .00 | .05 |
| 12. Trials V Tag E | −.14 | −.15 | −.09 | −.03 | −.16 | .06 |
| 13. Trials A Tag D | −.19 | .02 | −.02 | .01 | .00 | .03 |
| 14. Trials V Tag D | −.18 | −.14 | −.18 | −.02 | −.18 | −.09 |

*Note.* **p* < .01, ***p* <. 001

**Table S5.**

| Predictor | vMMN E | vMMN D | aMMN E | aMMN D |
| --- | --- | --- | --- | --- |
| AQ Social Skills | *F*(1,87) = 2.80, *p* = .098,  *η²* = .031 | *F*(1,87) = 2.42, *p* = .123,  *η²* = .027 | *F*(1,87) = 1.34, *p* = .251,  *η²* = .015 | *F*(1,87) = 0.03, *p* = .872,  *η²* = .000 |
| AQ Communication | *F*(1,87) = 1.58, *p* = .212,  *η²* = .018 | *F*(1,87) = 1.26, *p* = .212,  *η²* = .018 | *F*(1,87) = 3.38, *p* = .070,  *η²* = .037 | *F*(1,87) = 0.41, *p* = .523,  *η²* = .005 |
| AQ RIDO | *F*(1,87) = 0.59, *p* = .444, *η²* = .007 | ***F*(1,87) = 6.67, *p* = .011,**  ***η²*** **= .071** | *F*(1,87) = 0.57, *p* = .571, η² = .007 | *F*(1,87) = 0.93, *p* = .338,  *η²* = .011 |
| Negative SPQ | *F*(1,87) = 1.33, *p* = .252,  *η²* = .015 | *F*(1,87) = 2.07, *p* = .154,  *η²* = .023 | *F*(1,87) = 0.22, *p* = .643,  *η²* = .003 | *F*(1,87) = 2.13, *p* = .149,  *η²* = .024 |
| Disorganized SPQ | *F*(1,87) = 0.27, *p* = .606,  *η²* = .003 | ***F*(1,87) = 4.47, *p* = .037,  *η²*** **= .049** | *F*(1,87) = 1.18, *p* = .280,  *η²* = .013 | *F*(1,87) = 1.33, *p* = .252,  *η²* = .015 |
| Positive SPQ | *F*(1,87) = 2.97, *p* = .089,  *η²* = .033 | *F*(1,87) = 0.22, *p* = .642,  *η²* = .003 | *F*(1,87) = 1.78, *p* = .187,  *η²* = .020 | *F*(1,87) = 0.50, *p* = .480,  *η²* = .006 |

*Unified regression model including all AQ and SPQ subscales predicting auditory and visual MMN amplitudes across task difficulty conditions.*

*Note.* a – auditory, Autism-Spectrum Quotient – AQ, D – Difficult, E – Easy, SPQ – Schizotypal Personality Questionnaire, v – visual.
